# The Association between Green Space and Adolescents’ Mental Well-Being: A Systematic Review

**DOI:** 10.3390/ijerph17186640

**Published:** 2020-09-11

**Authors:** Yijun Zhang, Suzanne Mavoa, Jinfeng Zhao, Deborah Raphael, Melody Smith

**Affiliations:** 1 School of Nursing, The University of Auckland, Auckland 1142, New Zealand; jinfeng.zhao@auckland.ac.nz (J.Z.); d.raphael@auckland.ac.nz (D.R.); melody.smith@auckland.ac.nz (M.S.); 2 Melbourne School of Population and Global Health, University of Melbourne, Parkville, Victoria 3010, Australia; suzanne.mavoa@unimelb.edu.au

**Keywords:** green space, mental well-being, adolescent, urban planning

## Abstract

This systematic review summarised and evaluated the evidence for associations between green space and adolescents’ mental well-being. The PRISMA statement guidelines were followed for reporting systematic reviews. Fourteen articles met the inclusion criteria for this review. Synthesis suggests beneficial associations between green space exposure and reduced stress, positive mood, less depressive symptoms, better emotional well-being, improved mental health and behaviour, and decreased psychological distress in adolescents. Several studies found the relationship varied by demographic and socio-economic factors. The limited number of studies and the risk of bias were the main limitations, together with heterogeneity regarding green space and mental well-being assessments. Overall, this review highlights the potential contribution of green space in schoolyards. Improving the availability, accessibility and quality of green space is likely to generate positive impacts on adolescents’ mental well-being. More consistent evidence on the use of different types of green space and perceptions of features are needed in the future.

## 1. Introduction

Mental well-being is a fundamental component of health and quality of life, encompassing aspects of hedonic and eudaimonic well-being [1,2]. It is conceptualised as more than the absence of mental illness, and is protective for a range of health outcomes [3]. Among adolescents, mental well-being contributes to social, intellectual and emotional development, enriches self-esteem and academic achievement, and eases the transition into adulthood [4]. However, stress and anxiety levels are increasing among young people, putting them at risk of mental disorders and related co-morbidities, such as conduct and emotional disorders [5,6,7,8]. The World Health Organization estimated that between 10% and 20% of the world’s population of children and adolescents have mental disorders and problems, with half of all mental illnesses beginning by the age of 14 [9]. Decreasing rates of mental well-being and an increasing prevalence of early onset mental problems suggest the need for a better insight into determinants and triggers to enhance mental well-being among this age group.

Exposure to green space is a promising intervention for promoting adolescents’ mental well-being. A growing body of research has found that exposure to green space has a variety of positive impacts on young people’s health. These benefits include enhanced mental health and resilience [10,11,12], and increased physical activity and reduced risk of obesity [13,14,15]. It is evident that time spent in, or exposure to, green space can improve positive mood and emotions, provide a retreat from daily hassles, and reduce the risk of psychological and physiological stress in adolescents [16,17,18]. There is also evidence of lasting mental health benefits of green space exposure in childhood [19,20].

### 1.1. Measuring Green Space

The definition of green space varies across studies and lacks consensus [21]. Taylor and Hochuli [22] stated two possible interpretations of green space: either an area with water or vegetation, or urban public open space with vegetation. Green space exposure can be implied as contact between green space and a human [23].

It is worth noting that a range of approaches to measuring green space in the literature have been employed in studies exploring links between green space and health across a range of populations.

From the perspective of objective measures of green space exposure, studies can fall into two main categories. The first is assessments of availability, accessibility and visibility of exposure by how much green space there is in, close/access to, or can be seen from a specific location or area (e.g., home address, activity space) [24,25,26]. The green space metrics of this approach, such as Normalized Difference Vegetation Index (NDVI), percentage of green cover, are generally extracted from remote sensing image, land cover map and Google street view [27,28]. The second category is implementations of real time global positioning system (GPS) technology and geographic simulation to provide participants’ daily locations and movement patterns [28,29].

In addition to objective measures, some studies also employed measurements to tap into the subjective experience of green space. Survey questions about duration, frequency of visit and quality of green space are common ways to assess participants’ perceptions and experience [30,31]. Finally, experimental or quasi-experimental study designs have been used to explore the effects of green space exposure. Ideally, in such studies, the environment and activities in which they engage are controlled or evaluated in order to isolate the role of green space on mental health [32,33].

### 1.2. Green Space and Mental Well-Being

Previous systematic reviews have demonstrated relationships between green space and mental well-being across the course of life. Specifically, evidence shows that increased accessibility to green space and residing in areas with increased greenness are associated with improved perceived mental health [34,35,36,37]. Additionally, physical activity in natural outdoor environments has been linked with reduced negative emotions and fatigue [38,39].

A systematic review of 50 observational studies demonstrated associations between the amount of local-area green space and life satisfaction (hedonic well-being) in adults [21]. McCormick [40] reviewed the association between green space and the mental well-being of children and youth (aged 0–18 years) and found that access to nature was associated with mental well-being, attention, social support, behaviour, school performance and the management of attention deficit hyperactivity disorder (ADHD) symptoms. Tillmann, et al. [41] reviewed the relationship between mental health and accessibility to, exposure to and engagement with nature in children and young people (aged 0–18 years). Their findings show beneficial outcomes on all aspects of mental health, attention deficit disorder (ADD)/ADHD, overall mental health, stress, resilience and health-related quality of life (HRQoL). Similarly, Vanaken and Danckaerts [42] identified an association between increased green space exposure and reduced emotional and behavioural difficulties in children, particularly with regard to hyperactivity and inattention problems. For adolescents, the evidence from their study suggested a beneficial association with a reduced risk of depression. Importantly, beneficial associations were consistent across demographic and socio-economic variables. Most recently, Norwood, et al. [43] conducted a systematic narrative review of pre-post and longitudinal studies, concluding that passive exposure to nature promotes positive changes in attention, memory and mood in young people.

However, gaps remain in the evidence base. The variety of, and inconsistencies in, definitions and measures of green space mean that there is a lack of clarity on the features of green space important for young people’s mental health, and the measures that best represent these features. Furthermore, although there are a number of quality assessment tools to appraise risk of bias or the methodological strength of green space research, there is insufficient reporting of quality assessment in published systematic reviews specifically related to green space and adolescent mental well-being. Furthermore, no such reviews have focused on adolescents as a singular group of interest, with previous reviews combining child and adolescent population groups, limiting our understanding of the specific needs of this population group, and preventing definitive conclusions about the role of green space in adolescent mental health. Therefore, establishing and understanding an evidence-base for the effects of green space on the mental well-being of adolescents is vital. Therefore, the aims of this systematic review are: (1) to provide evidence for relationships between green space and adolescents’ mental well-being; (2) to appraise and to synthesise the evidence in relation to the quality of the studies included, and the consistency of results.

## 2. Materials and Methods

The systematic review was conducted following the Preferred Reporting Items for Systematic reviews and Meta-Analyses (PRISMA) statement guidelines (Table S1). The predefined protocol was registered (CRD42019141561) and is available on the PROSPERO website http://www.crd.york.ac.uk/prospero/.

### 2.1. Search Strategy

A bibliographic search of SCOPUS, GEOBASE, CINAHL plus (EBSCO Interface), Medline (Ovid interface), Cochrane central register of controlled trials (Ovid interface), EMBASE (Ovid interface), and PsycINFO (Ovid interface) was conducted by the first author (YZ) from January 2000 to October 2019. The databases were chosen and the search strategy was developed by all authors and adapted through consultation with a subject-specific research librarian. For the purposes of this review, mental well-being was defined as positive mental health, so search terms included: well-being OR “well-being” OR wellness OR “emotional health” OR “psychological health” OR “mental health” OR mood OR depress* OR anxiety OR stress OR happiness OR pleasure. Green space was defined as urban areas of vegetation [22], and search terms included “green space*” or greenspace* or greenness or greenery or “green area*” or “greenway*” or “green belt*” or “green corridor*” or “natural environment*” or “open space*” or park or parks or “natur* space*” or naturalness or garden* or playground or canopy or tree* or forest or forests or woodland* or “green roof*” or “roof garden*” or arboretum or “urban nature” or “green adj2 space*”. Adolescent was defined as people aged from 10 to 19 years old, based on the definition of adolescent of the World Health Organization [44] and the United Nations Children’s Fund [45], and search terms included teen* or adolescen* or youth* or juvenile* or “young people” or “young person” or “young adult*” or “high school*” or “secondary school*” or “senior school*”. For the type of study, we chose descriptive and observational research that tested the ability of green space to promote adolescents’ mental well-being.

The search terms were refined by considering previous related reviews [34,40,42], MeSH subject headings in Ovid, and expert comments and knowledge of the research team. Keyword searches of article titles and abstracts were categorized into three domains: (1) green space, (2) mental well-being, (3) adolescents. An example of a search strategy in Ovid is available in PROSPERO (CRD42019141561) and a full search strategy is in Table S2.

### 2.2. Eligibility Criteria

Studies were eligible if they met criteria relating to green space, mental well-being outcomes, participants and type of study, as shown below:

- Studies were included if they included a publicly accessible open space including greenery, such as areas with plants, including forests, parks, gardens, and woodlands.
- Studies with a mix of green space (e.g., green and blue space) were included if green space was analysed and reported separately.
- Studies used an objective (e.g., land cover maps, remote sensing data) or subjective (e.g., standardized questionnaires) measure for quantity or quality of green space, or a clear description of the green space, e.g., in a school or residential environment.
- Exposure to green space measure was assessed during adolescence, so studies that measured historical exposure (e.g., during childhood) without current exposure (during adolescence) were excluded.
- Mental well-being outcomes included any of the following: mood, stress, anxiety, depression, happiness, pleasure, emotional health, psychological health, and mental health.
- Participants had to be adolescents aged 10–19, or if ages were not reported, school level had to be reported as high school, secondary school, junior high or intermediate school.
- Studies with mixed age groups were included if findings for the adolescents were reported separately (i.e., stratified by age group).
- Descriptive and observational studies with either a cross-sectional, experimental or longitudinal design or randomized controlled trials or intervention studies were eligible.

In addition, this systematic review was limited to peer-reviewed articles, available in full-text, English language, and published from January 2000 to January 2020. This time limitation was set as the concept and measurement of green space is rapidly evolving, and most of the relevant literature has been published during the last two decades.

Studies were excluded if: (1) they examined the impact of green space using hypothetical scenarios, (2) their study population had known medical conditions (e.g., asthma), or (3) were qualitative, systematic reviews, expert opinions or conference proceedings.

### 2.3. Study Selection

Initial reviewing and identification of titles and abstracts was undertaken by the first author (YZ), and 10% of the titles and abstracts were randomly selected to be screened by a co-author (DR). Full texts were obtained for all titles and abstracts that appeared to meet the inclusion criteria. YZ then screened the full-text articles and assessed their eligibility for inclusion, and 10% of the selected full-texts were also screened by DR. Discrepancies between the review authors in selection processes were resolved through discussion and reasons for excluding studies were recorded. Protocols were updated and applied to the 100% screening process. No inter-rater reliability testing was undertaken as the process allowed for refining and improving the search criteria which was then applied to the entire process.

### 2.4. Data Extraction

Key data were extracted from each eligible article into a study-specific data collection form in Microsoft Excel, which was generated from a previous systematic review on green space and mental health by Gason et al. [34]. The form includes study location, authors, publication year, study design, population description, statistical methods, green space variables/definitions, green space calculation/measures, outcome variables, outcome measures, co-variables of adjustment and mediation, and key findings.

### 2.5. Methodology Quality Appraisal and the Strength of Evidence

The quality of included studies was evaluated using the Lachowycz and Jones [46] methodological quality assessment tool as employed in the previous similar review [34]. The original quality assessment tool was adapted to improve its suitability for assessing articles included in this review, and to generate an overall appraisal of each study as outlined in Table S3. The quality score was based on 11 different items which could be scored as 0, 0.5, or 1, with a higher score indicating higher quality for that variable. The original tool used a binary score of 0 or 1; however, in this review, an additional score of 0.5 was used in situations where there was insufficient evidence to score the item with confidence. For each study, the total quality score was calculated by summing the scores across the 11 items. YZ completed a quality appraisal of each study included, and 10% of the selected studies were also assessed by a review author (MS). Reviewers resolved disagreements by discussion and an arbitrator (SM) adjudicated unresolved disagreements.

In order to facilitate the classification of the strength of evidence for a causal relationship between green space and adolescents’ mental well-being outcomes, a category for inconsistent relationships due to different green space and mental well-being measures was added.

### 2.6. Summary of Findings

A formal meta-analysis approach was judged inappropriate for this research because of the heterogeneity of green space and mental health outcome measures. A narrative synthesis was undertaken to summarise study characteristics and their findings.

## 3. Results

**Figure 1:**
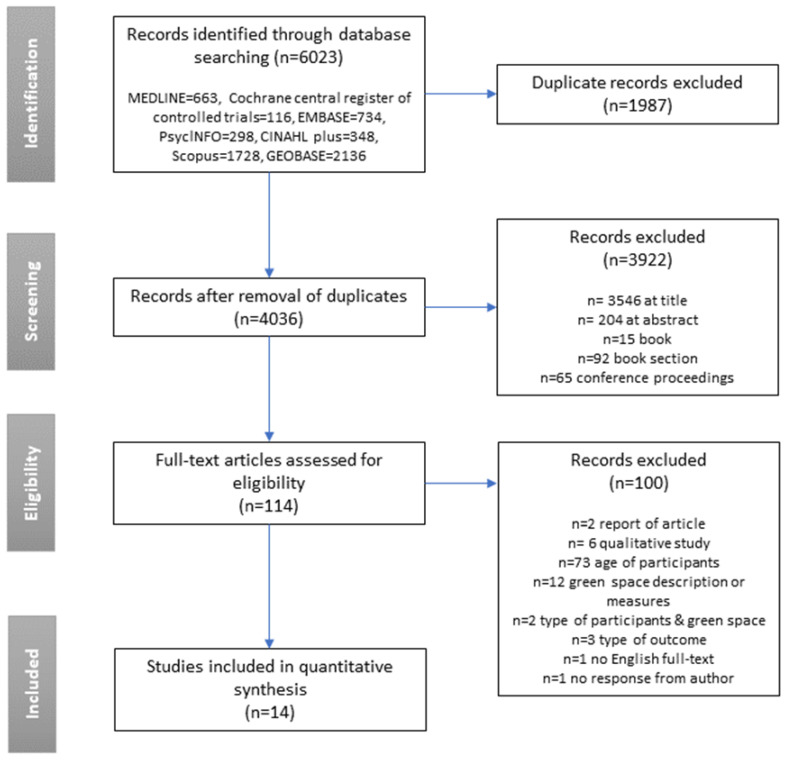
Article selection process.

Figure 1 provides the flow diagram of the articles included and excluded from the review. From the seven databases, 6023 articles were identified. After discarding 1987 duplicates, 3922 were excluded at the title or abstract screening stage, and 114 were assessed in full text. In total, 14 articles met the inclusion criteria for this review.

Key characteristics of the included studies are presented in Table 1. Most of the studies were cross-sectional (N = 10), followed by controlled experiments (N = 3) and one longitudinal study. Five of the 14 articles were conducted in the United States, with the remainder from the Netherlands (N = 2), the United Kingdom (N = 2), Canada, Germany, Austria, Australia and Aotearoa New Zealand (all N = 1). The sample size of the study populations ranged from 60 to 17,249 participants.

### 3.1. Quality Assessment and the Strength of Evidence

The results of the methodological quality assessment are provided in Table S4. All in-scope studies scored equal to or above 7/11 according to the appraisal. Assessment criteria where the majority of studies were deficient include: no consideration of the type of green space (N = 7), no measure of green space use (*N* = 13), and analysis at an ecological, rather than an individual level (N = 6).

Overall findings for relationships between green space and mental well-being are presented in Table S5. Eight of the 14 studies reported a significant positive relationship between green space and adolescents’ mental well-being, whereas four findings were deemed non-significant. Another two studies reported inconsistent results due to different green space and mental well-being outcome measures.

### 3.2. Mental Well-Being Outcomes

Mental well-being outcomes were assessed by a range of different approaches and were captured through variables such as stress, mood, depression, emotional well-being, mental health (well-being) and behaviour and psychological distress. Findings varied widely but the association between green space and mood and stress was the most consistent. Three studies reported beneficial associations between green space and mood [28,47,48] and three of the four studies that explored the relationship between green space and stress found a significant association with stress reduction [32,49,50].

The remaining studies predominantly showed beneficial relationships between green space and depression, emotional well-being and mental health and behaviour. One study showed a beneficial association between green space and depression reduction [51], one between green space and emotional well-being [29], one with mental health and behaviour (using the strengths and difficulties questionnaire; SDQ) [52]; however, three showed a non-significant relationship [53,54,55]. A further study reported a positive association between green space and reduced risk of serious psychological distress [56].

### 3.3. Covariables, Mediators and Moderators

**Figure 2:**
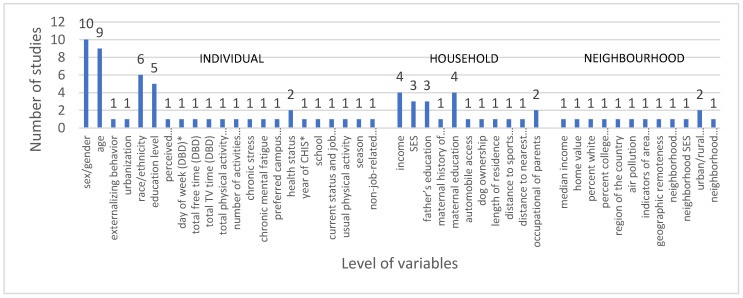
Covariate, confounders, moderators and mediators.

A multitude of covariables, moderators and mediators in the association between green space and mental well-being were used in the fourteen studies. Forty-five different covariates and confounders, nine moderators and three mediators were identified in this review. The most common covariables were sex/gender, age, race/ethnicity and socioeconomic status (SES) (Figure 2). Age, gender, ethnicity, physical activity and stressful life events were included in moderation models. Examinations of the moderating effects were uncommon and largely non-significant. Studies reported no clear pattern in the moderating effects of age, gender, ethnicity, and stressful life events. Greenwood and Gatersleben [47] indicated that ‘being with a friend’ considerably increased positive affect of outdoor green space on adolescents. One study showed that ‘physical activity and percentage park area’ did not predict perceived stress [50]. However, the Ward et al. [29] study found that emotional well-being was in part attenuated by separately adjusting for physical activity. Three studies in this review did not detect evidence for the mediation role of ‘perceive improvement and use of greenery’ [53], social cohesion [56] and physical activity [49].

### 3.4. Green Space Measurement

#### 3.4.1. Amount of Greenspace

The definition and measure of green space varied widely. Eight studies, one of which was a laboratory experiment, explored the relationship between quantities of local-area green space and adolescents’ mental well-being. Five cross-sectional studies examined associations between neighbourhood greenness and adolescents’ mental well-being. Two of the five studies quantified greenness as the percentage of green space for each postal code area or UK ward in the adolescents’ residential area [33,55], and three used a Normalized Difference Vegetation Index (NDVI) derived from satellite images in buffers of 250–1250 m, 250–950 m or 500 m around the residential addresses of participants [49,51,56]. One study measured the percentage of green space within each 5 km radius circular buffer in school settings [54], and another used neighbourhood park access by measuring the area of park land divided by total land within 0.80 km distance (along street networks) of a participant’s home [50].

Weeland et al. [33] conducted a laboratory study, measuring adolescents’ respiratory sinus arrhythmia (RSA) activity, which captures changes in heart rate during stress and recovery. They found that neighbourhood greenness was not related to RSA rest, reactivity and recovery, and was not a predictor for RSA recovery. Using the strengths and difficulties questionnaire, Mueller et al. [55] found that there was no significant relationship between the percentage of green space in wards (a division or district of a city or town) and well-being in 3683 adolescents after controlling for cofounders.

Three studies found evidence of a positive relationship between high NDVI (high levels of greenness) and adolescents’ mental well-being. Bezold et al. [51] reported that higher greenness (using a 1250 m radius around residence) was associated with lower depressive symptoms. The association was stronger in middle school students than in high school students, although the difference was not statistically significant. Similarly, Herrera et al. [49] found the prevalence of high levels of job-related chronic stress decreased with increasing level of greenness in a 500 m radius around the homes of 2690 adolescents aged 16 to 18 years in Germany. Wang et al. [56] found that an interquartile increment of NDVI in a 350 m residential buffer predicted decreased serious psychological distress (SPD) among teens in the United States.

Huynh et al. [54] reported a weak correlation with green space (percentage of land features within a 5 km radius circular buffer around schools) and positive emotional well-being. Findings suggested that demographic characteristics, family affluence, and perceptions of neighbourhood surroundings might be stronger potential determinants of emotional well-being than school neighbourhood green space.

One study investigated the association between perceived stress among adolescents and access to parks [50]. The findings show that residing in neighbourhoods with high park access was linked to lower stress.

#### 3.4.2. Experimental Studies

Three studies explored the effects of different green spaces on adolescents’ mental well-being, including one study that compared a green space to a non-natural control to highlight the effects of green space [32,47,48]. These studies found that green space in/around school (i.e., classroom window views to green space) benefits to adolescents’ mental well-being outcomes, including improved momentary mood state [48], positive affect [47] and reduced stress [32]. No significant association between green space and improved attentiveness was found [47].

Wallner et al. [48] measured the effects of spending a study break in three different urban green spaces (small park, larger park, forest) on the mood of sixty students from three schools in Vienna. Positive associations between three types of green space and mood were found. A reduction in mood on returning to class was less pronounced for those in the forest than for those in the small and large urban parks. However, the study lacked an indoor control group, which means it is unclear if the improved mood was due to the break or the time spent in green space. Li and Sullivan [32] compared different views from windows in three classrooms (no window, barren window and green window) in a randomized controlled experiment with 94 high school students in the United States. The results demonstrate that classroom views onto green space increased students’ recovery from a modified Trier Social Stress Test [57]. Another field experiment, with 120 participants aged 16 to 18 years in the United Kingdom, examined the impact on mood of being in a green space at school compared with being in an indoor environment [47]. Participants reported an increase in positive mood in the outdoor environment compared with a reduction in mood in the indoor environment. Participants also reported that being with a friend considerably increased the positive affect in green space.

#### 3.4.3. Implementation of Technology

Recent technological improvements have enabled the collection of objective, high-resolution data on individuals’ daily activities. In two studies, GPS tracking and environmental exposure assessment were employed to measure green space exposure. Li et al. [28] presented a cross-sectional study that used GPS to track the daily activities of 155 high school students over four consecutive days. The concentration of nature that participants were exposed to was calculated using an algorithm to assess Google Street View images for the locations that participants visited. Significant associations were found between the concentration of nature exposure and the daily mood of adolescents. Another study in Aotearoa, New Zealand used data from GPS facilitated sports watches to measure time spent in green spaces and on physical activity [29]. Spending time in green space was related to higher levels of physical activity and better emotional well-being, as assessed using the Life Satisfaction Scale (LSS) [58], the Ten Domain Index of Well-being (TDIW) [59] and a single item measure of happiness with life as a whole (HS).

#### 3.4.4. Mix of Green Space Measures

Only two studies assessed both perceived and objective measures of green space. Feng and Astell-Burt [52] assessed green space quantity (percentage of green space land use) and self-reported green space quality. Higher green space quality was associated with lower emotional symptoms, conduct problems, hyperactivity and peer problems, but there were no associations with the quantity of green space.

Only one study was conducted specifically in a low socio-economic status neighbourhood [53]. This study used a longitudinal design to assess the impact of perceived and objective changes in neighbourhood greenery on depressive symptoms and physical activity of adolescents. Perceived greenery was measured by questionnaire and interview while the objective information of greenery interventions (type, duration, scale) were collected. The findings show a non-significant trend towards associations between perceived and objectively assessed increased greenery and declining depressive symptoms in adolescents.

### 3.5. Risk of Bias Across Studies

Overall, the individual study methodological quality assessment showed high quality in study, limiting the ability to consider findings in relation to study quality. As with all systematic reviews, it is possible that publication bias and selective reporting within studies occurred. However, a number of studies included reported non-significant results, suggesting a lower risk of publication bias across studies in this review. No evidence of selective reporting with specific regard to mental well-being and green space analyses was observed in this review.

### 3.6. Summary of Findings

The most common setting for studies included in this review was residential green space and most employed a cross-sectional study design. Only one study combined quantity and quality of green space. Generally, greater amounts of green space appeared to have a positive impact on, or association with, adolescents’ mental well-being, although four studies did not find significant relationships and two reported inconsistent associations.

## 4. Discussion

This systematic review summarised the current state of research on the benefits of green space for adolescents’ mental well-being. It focused on the effects of green space, by excluding studies that had other integrated components (e.g., gardening or after school gaming). It also sought to determine the potential for green space to promote change by including experimental studies. Following PRISMA protocols, relevant results were systematically identified, screened for eligibility, underwent data extraction, and were assessed for methodological quality. As a result, fourteen studies which covered a wide range of adolescents’ mental well-being and green space aspects were included.

### 4.1. Specificity of Green Space and Mental Well-Being Research among Adolescents

Synthesis of the literature provided evidence of the positive benefits of green space for adolescents, especially in terms of reduced stress, positive mood, less depressive symptoms, better emotional well-being, improved mental health and behaviour, and lower psychological distress. In line with previous systematic reviews conducted for adults and children [21,34,35,36,37,40,41,42], a majority of findings showed statistically significant positive relationships between green space and mental health.

### 4.2. Green Space Measures

Studies demonstrated that higher NDVI with a 1250, 500 and 300 m buffer surrounding the home address had associations with lower depression, stress and SPD [20,49,56]. This finding aligns with a New Zealand study published after the search, which found significant relationships between reduced depressive symptoms in adolescents and increased mean greenness (NDVI) in residential neighbourhoods (defined as 400 and 800 m buffers around meshblock boundaries) [60]. Authors also reported a negative association between variability in greenness in 1600 m buffer and adolescents’ well-being. In contrast, no significant relationships were reported in two studies that used the percentage of green space in neighbourhoods using administrative area boundaries (postal code and ward) [33,55]. The different results might be due to the choice of neighbourhood definition. As with many other outcomes, such as physical health, the choice of boundary can lead to different results [61,62,63,64]. Studies exploring different health outcome measures have also raised concerns about inconsistent buffer distances and types (e.g., Euclidian or network). Studies focused on mental health were reported to employ smaller buffer distances, compared to physical health research [26]. There is no consensus to what buffer distance is optimal to define the exposure area [27]. According to the results of this review, a distance between 300 and 1600 m is suggested for future adolescents’ mental well-being related research. Meanwhile, in order to facilitate the understanding of which spatial exposure provide better results, it is important to consider employing more than one buffer in future research. Further, it is worth noting that most of the selected and effective buffer distances in this review do not align with buffer distances used in adult or child mental health studies, with one systematic review showing that these tend to be within 300 m (with a median value of 400 m) [26]. More research is required to understand what buffer sizes are most appropriate to understand key associations between green space and health in adolescents, as compared with other population groups (e.g., younger children or older adults). Moreover, it is not known what key characteristics of green space might be more or less attractive to this population group. Another possible reason that leads to the differences between greenness (NDVI) and the percentage of green space may be partly due to differences in the quality of green space. Green space quality can be assessed subjectively [30] or via objective assessment [65,66]. Quality can include a variety of aspects of green space, of which vegetation is one, and it is possible that NDVI captures an aspect of quality.

Middle and high school students spend large amounts of time at and around schools, yet only three studies in this review examined the impact of school-related green space on students’ mental well-being [32,47,54]. One study [54] did not detect a relationship between green space quantity in school neighbourhoods and positive emotional well-being. A study published after the review date found a similar null relationship between school-based greenness using mean NDVI and students’ mental health [67]. It is possible that the amount of green space around a school may be less relevant to mental health than other characteristics of green space, such as type, quality, and usage [31,68]. Similarly, views of green space in school yards may also be more relevant than greenspace quantity since the two experimental studies of window views of green landscape and outside natural environments demonstrated lower stress and increase positive affect, respectively [32,47], aligning with evidence that green space in schoolyards may be capable of promoting improved physiological well-being and reducing physiological stress [69]. Another possible reason is that the academic stress or peer relationships may outweigh mental health benefits [70,71]. The results of this systematic review emphasise the potential importance of green space in school settings. Future studies to explore stressors that factor in adolescents’ mental health and find promising ways to ease negative impacts are encouraged. Furthermore, exploring whether the potential positive effects of green space (using a range of green space measures) in school settings and school neighbourhoods are needed to provide designers, planners, and policymakers with evidence to enhance student well-being through school yard and school neighbourhood design.

Currently, there is no standardised approach to define green space or to measure it, and definitions and measures sometimes overlap. Meanwhile, the definition of green space in research might not be able to represent actual green space exposure comprehensively. When a definition is provided, there is variation in what is meant by the term green space [22], such as surrounding greenness which is measured by NDVI in a certain buffer as a marker of green space exposure in some research. However, green space is generally comprised of vegetation and is associated with natural elements [22]. In this review, four types of measures were used in the fourteen studies: the amount of green space, experimental studies, new technology implementation and a mix of measures. The diverse definitions and measures used in the studies included in this review potentially lead to different results and conclusions.

However, these inconsistent results also contribute to the question as to whether the green space measure appropriately represents green space. Green spaces can be subdivided into publicly accessible and private green space; or into natural, agricultural and urban green space [72]. The objective and subjective measures may assess different aspects of green space [53]. The approaches used to measure green space are at the developmental stage. Most studies used objective measures of green space, but few studies used both subjective and objective measures [52,53]. The lack of subjective measures might lead to missing predictors of mental well-being. The combination of both measures of the environment was previously recommended [73]. Regarding experimental or quasi-experimental study, environmental settings are likely different from the actual daily green space exposure in adolescents. Such a mismatch may lead to inconsistent results of green space’s benefits on mental health. Therefore, robust study designs are needed in order to capture adolescents’ mental health in everyday exposure.

Future research would ideally measure adolescents’ potential and actual access to green space, as well as their experiences of green space. However, this is challenging. The most widespread approaches to assessing green space use available land cover datasets, satellite images, or aerial photographs [74,75,76] are susceptible to the modifiable areal unit problem (MAUP) [77]. Varying scales or shapes are employed to represent and measure spatial zones leads to the statistical bias. The MAUP refers to two related problems. One is the zoning effect, which implies that drawing the boundaries of geographical areas differently at the same scale may lead to different results. The other is the scale effect, which may produce different results when utilising differently sized geographical areas [27,78,79]. Two studies employed new technology such as GPS tracking, which offers an opportunity to examine in detail surrounding green space and represent adolescents’ true activity space that exerts contextual influence on mental well-being [28,29]. The combination of GPS tracking and experience sampling method has been promoted as an area of future research, as it allows researchers to objectively analyse the environments adolescents’ are exposed to and couple their environmental exposure with subjective feelings and evaluations (e.g., using participatory geographic information systems) [80,81]. Given the limitation of GPS technologies so far, such as signal loss and slow location detection [82], ecological momentary assessment (EMA) may prove a useful strategy [83]. EMA involves sending short questions to participants, usually via cell phone, to capture key measures of interest (e.g., where are you? what are you doing? who are you with? how do you feel?). This approach allows researchers to collect accurate activity and location data and real-time assessment of mental well-being.

Finally, more longitudinal studies are needed to explore the associations between mental well-being and green space, even though they may suffer from a lack of change in predictor variables (e.g., the average change in greenness during the years is unpredictable) [53].

### 4.3. Potential Mediators, Moderators and Confounders

Aside from green space directly affecting adolescents’ mental well-being, green space also has indirect impacts on adolescents’ mental health. Theories and recent studies from adults indicated that the perception of green space, physical activity and social cohesion can mediate the relationship between green space and mental well-being [30,84,85]. In adult research, evidence has suggested that social cohesion at least partially acts as a mediator in the relationship between green space and mental health [86,87,88]. In contrast, the study included in this review found that social cohesion did not mediate the green space–mental well-being relationship [56]. Similarly, no significant mediation role was reported for physical activity in this review, which is in line with some previous adult findings [87,89,90] but not with others [88,91]. Overall, the findings from this review are consistent with another youth study, which revealed no mediation for social cohesion and physical activity between objective green space and mental health [92]. The inconsistent results may be partly due to different measures of mediators, green space and mental health, and to the different mechanisms operating at different stages of adolescence. Another possibility is the use of electronic devices among adolescents. The screen time of adolescents might reduce their time partaking in physical activity [93], attention and perception of physical space, and face-to-face communication with people.

Issues of access and equity are also of concern. Socioeconomic status is one of the main confounding sources between adolescents’ mental well-being and green space. Young people from disadvantaged families tend to have poorer well-being and reside in neighbourhoods with a lower quantity or quality of green space. This is supported by research showing that participants living in socially disadvantaged areas were more likely to have the lowest strata of green space quantity [52,94,95]. Li et al. [28] found that adolescents living in low income neighbourhoods/households have less exposure to vegetation in their accessible environment than their peers from medium and high-income households. As a result, they tend to report limited green space engagement and less frequent visits [96]. The imbalance in green space exposure is an important equity issue. Questions arise such as if more deprived neighbourhoods have less green space, and does this situation present in every country? What types of barriers prevent residents from accessing green spaces? What spatial, built environment, mobility and social constraints might lead to these inequities? What measure can be applied to address the imbalance? Additional analyses are needed to explore adolescents’ patterns of activity in green spaces and their preferences for green space features, in order to develop initiatives that improve equity and access to green space.

This review identified factors influencing the relationship between green space and the mental well-being of adolescents and potential mediators and moderators of the relationship. Although the evidence is limited and results are mixed, some findings are consistent with previous reviews, which suggest potential partial mediation of the green space–mental health relationship via physical activity, reduced air pollution and social interaction in children and young people [42]. However, the available evidence on the role of potential mediators and moderators of the health effects of green spaces in adolescents remains scarce. Future studies are encouraged to identify potential mechanisms by which green space may (or may not) boost mental well-being using robust study designs that adequately account for confounding, moderating and mediating factors.

### 4.4. Critical Review of Studies’ Quality

The results of this systematic review highlight the need for more evidence on adolescents’ perceptions and use different types of green space, green space affordances, and how physical and psychological development explains in their choices that subsequently influence green space exposure and mental well-being. Matsuoka [97] indicated that not all types of green space are beneficial for high school students and some studies have shown that benefits depend on the quality of green spaces [38,87,98]. The positive impact of the type, use and perception of green space on residents mental health was convincingly demonstrated in previous studies [99,100,101]. Furthermore, research on adolescents’ favourite places proposed that adolescents can retreat to places such as stores, shopping malls, streets and sports facilities [102,103,104]. There is a need for future studies to explore the importance of green space within such “third places” [105] in adolescents’ mental well-being. Accordingly, mixing land use is likely to be a promising approach to bring green space-mental health benefits to adolescents’ favourite places. In doing so, public green space can be designed with different functions, such as bring in outdoor gym facilities, and be accessed with other “third place”.

### 4.5. Implications

#### 4.5.1. Small Scale Green Space

The current review calls attention to the essential impacts of neighbourhood-level green space on promoting adolescents’ mental well-being. One feasible approach that can facilitate this is to formulate small green space design guidance that responds to the needs of young people. Previous studies have proposed that nearby natural environments and neighbourhood-level interventions are likely to promote mental health [89,106]. Small-scale space such as rain gardens within the street, pocket gardens, communal roof gardens and soft landscaping in front of or along the front of constructions are recommended for future urban green space design, which may create daily exposure and bring mental health benefits.

#### 4.5.2. Biodiversity

Green spaces can support biodiversity, which may have mental well-being benefits [107]. While our review did not identify studies that assessed biodiversity, emerging evidence suggests that biodiversity is related to adults’ well-being [108]. Therefore, more practical design approaches are required to protect rare and vulnerable habits and species. In this respect, green corridors and reserves are promising approaches that can be used to extend and enhance existing ecosystems.

#### 4.5.3. Multi-Functional Green Space

Green space has more than one function, such as combining learning, working, leisure and entertainment together, which is encouraged in future design. Since green space can be the third place to enhance adolescents’ mental health [102], transforming and reconstructing the function of green space is a possible way to meet adolescents’ needs. Traditionally narrow/siloed categorisations of space could be blurred to encourage multiple functions and use of differing spaces. For example, learning and shopping activities can be expanded to green space. The forms designed for adolescents, such as outdoor study rooms, shared study cabin or street markets, are options for future spatial design.

#### 4.5.4. Adolescents’ Participation

Benefits may be gained when urban designers and policy makers take adolescents’ views into account when endeavouring to improve their mental well-being. In this respect, apart from survey questions, adolescents could participate in the design process with the support of emerging technologies in future design or research. For example, wearable devices and mobile internet can be used to support and share real-time records and collect adolescents’ daily emotions and motions, face-to-face social activity and routines of daily activity. Furthermore, sensing facilities and databases could be used to establish intelligent information systems, adolescents could use the app to control virtual reality (VR) to transform the context based on their preference. Finally, participatory and co-design processes that involve adolescents in planning may be important. Such approaches align with the United Nations Convention Right of the Child [109]. Co-benefits of such approaches may also include youth development, improved self-efficacy to participate in civic processes, and lay a foundation for environmental stewardship [110].

### 4.6. Review Limitations and Future Research

Several limitations need to be considered when interpreting the findings of the present review. First, this systematic review did not include non-English language articles, which may in part explain the underrepresentation of studies outside English language regions. All of the studies included in this review were conducted in developed regions such as the United States, United Kingdom, Australia, New Zealand and Europe, and its findings are limited accordingly. Inconsistent findings from previous international studies and the lack of studies from developing countries indicate a need for more research from more regions. Development of green space remains a challenge for cities in the developing countries [111]. Therefore, in order to further understand the benefits of green space for adolescents’ mental well-being across countries and establish a global framework, future research in developing countries and in a variety of culture background is warranted.

In addition, this review focuses on published peer-reviewed journal articles; grey literature and unpublished articles were not included. These limitations may affect the generalisability of the findings. Third, we excluded qualitative studies that also evaluated beneficial health effects of green space. The inconsistent associations across studies may be related to the different definitions and measurements of green space and mental well-being. Individuals’ subjective perception of green space also vary across sociocultural contexts [30]. In the interest of providing more consistent, clear and direct information to inform policy and practice based on the evidence of the effects of exposure to green space, we chose to focus on quantitative studies in this review. Future reviews may consider focusing on qualitative studies only, or both qualitative and quantitative. Furthermore, outcomes related to mental disorders such as cognitive problems were beyond the scope of this review. Since mental well-being is an umbrella concept, the exclusions were taken in the interest of generating epidemiological findings that could be translated into public policy and practice.

Meta-analysis was difficult to conduct due to the heterogeneity in research design, limiting the understanding of the characteristics or measures of green space that are optimal for adolescents’ mental well-being. With the increasing number of studies in this field, more standardised methods and protocols are needed to support meta-analysis in future green space-base studies [26]. For example, utilising frameworks such as the Spatial Lifecourse Epidemiology Reporting Standards (ISLE-ReSt) statement [112] is a possible way to improve reporting. Such approaches help to make reporting more consistent in research and facilitate meta-analyses where appropriate.

There are a range of measures that could be considered proxies for mental health in adolescents, or could be related in some important way; however, this review focused solely on studies that included direct measurers of mental well-being. It would be worthwhile for future research to consider factors such as academic achievement [113,114,115], cognitive development [116] and attention [117].

## 5. Conclusions

In conclusion, the body of literature examining green space and adolescents’ mental well-being is limited, shows mixed results, and is dominated by cross-sectional studies. We found sufficient evidence to warrant further research on the importance of green space for the well-being of adolescents. This review offers insight into the links between green space and adolescents’ mental well-being through different measures. Combining objective and subjective measures will provide a more comprehensive and accurate assessment to understand the relationship and mechanism. These two measures should not be used interchangeably. Consideration of confounding, moderation, and mediation pathways should be the focus of future investigations.

For policy makers, urban planners and public health researchers, it is important to understand how and where to design, preserve or restore certain types or quality of green space rather than aggregate and homogenize different spatial typologies [101]. In this respect, green space measures could consider include green space type, use and perceptions rather than only measuring quantity within an area. Further exploration of the unique characteristics of forest settings is needed to understand their role in mental well-being promotion. Innovative urban design approaches could seek to imitate key forest characteristics of importance in urban contexts. Indicators of minimum thresholds for green space requirements, such as ratio, type, quality of green space, could be provided when designing, planning and renovating urban areas at the neighbourhood level.

The present findings call for the provision of sufficient green space to protect the mental well-being of adolescents in an urbanising world.

## Figures and Tables

**Table 1:** Main characteristics and results of the studies on green space and adolescents’ mental well-being.

NO.	Author, Year, Study Location	Study Design	Population Description	Statistical Methods	Green Space Definition	Green Space Calculation/Measure	Mental Well-Being Outcome	Outcome Instrument	Covariates of Adjustment, Moderator and Mediator	Key Findings
1	Weeland et al. 2019 (Netherlands) [33]	Laboratory session in a longitudinal study	*n* = 715, Mean age = 16.3 years	Multiple regression analysis	Neighbourhood greenness was characterized as public green space (i.e., open green space or parks)	Greenness was assessed as the percentage of adolescents’ neighbourhood area (using the postal code of the participants)	Stress reactivity and recovery	Respiratory sinus arrhythmia (RSA)was operationalized as the heart rate variability in the high-frequency band (0.15–0.40 Hz).	Covariates: sex, age at T1, externalizing behaviour at T1 (CBCL), urbanization, and socio-economic status (SES; income, education, and occupational level of parents). Moderator: stress life events	Neighbourhood greenness at T1–T3 was not related to RSA rest, reactivity and recovery at T3, and it is not a predictor for RSA recovery (B = 0.03, SE = 0.04, *p* = 0.48, 95%CI: −0.05–0.12)
2	Bezold et al. 2018 (United States) [51]	Cross-sectional study	*n* = 9385, aged 12–18 years old	Logistic regression models Generalized estimating equations	Neighbourhood greenness	NDVI in 250 m and 1250 m buffer surrounding a subject’s residence	High depressive symptoms	McKnight Risk Factor Survey (MRFS)	Covariates: individual (self-reported race/ethnicity, grade level, age, and gender), household (income, father’s education, and maternal history of depression), and neighbourhood (median income, home value, percent white, and percent college educated, region of the country, air pollution)	An interquartile range higher peak greenness in the 1250-m buffer was associated with 11% lower odds of high depressive symptoms (95% Cl 0.79–0.99). This association was not statistically significant but stronger in middle school students than in high school students.
3	Feng et al. 2017 (Australia) [52]	Cross-sectional study	*n* = 3083, aged 12–13 years old	Negative binomial regression	Neighbourhood green space exposure Quantity of green space (greenness) Quality of green space	Percentage of land-use within each SA2 of residence covered by green space Statement of neighbourhood green space quality	Mental Well-being	Goodman’s 25-item Strengths and Difficulties Questionnaire (SDQ) Total difficulties score (TDS) (emotional symptoms, conduct problems, hyperactivity and peer problems scales) Internalizing subscale (emotional and peer symptoms scales)	Confounders: socioeconomic circumstances, indicators of area disadvantage, geographic remoteness, maternal education, child age and gender	Lower mean TDS scores were significantly lower for participants living near good quality green spaces for child-reported TDS (RR 0.871, 95% CI 0.809 to 0.938). internalising subscale was statistically significant associate with green space quality (RR 0.855, 95% CI 0.777 to 0.940)
4	Feda et al. 2015 (United States) [50]	Cross-sectional field study	*n* = 68, aged 12–15 years old	Multiple regression	Park area of residence (nature trails, bike paths, playgrounds, athletic fields and state-, county- and town-owned parks)	Park access: the area of park land divided by total land within 0.80 km distance (along street networks) of a participant’s home	Perceived stress	Perceived Stress Scale (PSS)	Covariates: SES, usual physical activity Moderator: gender, usual physical activity	Percentage of park area (β = −62.573, *p* < 0.03) predicts perceived stress among adolescents. Access to neighbourhood parks buffers adolescents against perceived stress after controlling for socio-economic status and physical activity. Usual physical activity as a moderator term was not significant (*p >* 0.05) The interaction term of ‘gender’ and percentage park area’ (*p >* 0.13) did not predict perceived stress.
5	Greenwood et al. 2016 (United Kingdom) [47]	Field experien-ce	*n* = 120, aged 16–18 years old	Mixed between- within subjects analyses of variance with follow-up *t*-tests	Outdoor environment was a peaceful grassed quadrangle surrounded by the school building on all four sides, but with a high degree of greenery, including a number of large trees, shrubs and flowers.	NA	Mood	Zuckerman’s (1977) Inventory of Personal Reactions (ZIPERS)	Moderator: ‘alone’, ‘with a friend’, ‘playing a game on a mobile phone’	Taken across all contexts, there was a significant interaction effect for environment, with teenagers reporting an increase in positive affect in the outdoor environment containing natural elements (Mpre = 11.48, SD = 3.20, Mpost = 12.57, SD = 3.58) compared with a reduction in positive affect in the indoor environment (Mpre = 11.03, SD = 4.09, Mpost = 10.75, SD = 4.26; F(1114) = 7.68, *p* = 0.007, partial eta squared = 0.06), being with a friend considerably increased positive affect. there was no interaction effect for either environment or context for attentiveness
6	Gubbels et al. 2016 (Netherlands) [53]	Longitudinal study	*n* = 994 aged 12–15 years old participated in the first measurement wave (May 2010-May 2011). *n* = 401 filled in the question-naire for the second measurement between May and July 2012.	Paired sample t-tests Bivariate correlations Multi-level linear regression analyses Methods of baron and kenny for mediation	Residential greenery	Perceived greenery: The Neighbourhood Walkability Scale Greenery interventions: changes in objective (type, duration and scale) and subjective (standardized questionnaires and extensive face-to-face interviews) amount and quality of greenery	Depressive symptoms	The Center for Epidemiologic Studies-Depression Scale (CES-D)	Covariate: demographic and socioeconomic covariates (gender, age, ethnicity and educational level), season Mediator: perceived improvement and use of greenery in the living environment	In the whole sample (20 districts), changes in the number of trees and nature in the neighbourhood were not significantly related to changes in depressive symptoms in adolescents (CES-D; β = −0.03, *p* > 0.05); As regards the sample from the District Approach (10 districts), objective improvements in perceived greenery had non-significant associations with a decrease in adolescents’ depressive symptoms.
7	Huynh et al. 2013 (Canada) [54]	Cross-sectional study	*n* = 17,249, students grades 6 to 10, mostly ages 11 to 16 years	Multilevel logistic regression	Nature space only contain land feature around school	The percentage of total land within each 5 km radius circular buffer surrounding each school that consisted of land feature, the buffers were divided into equal quartiles based upon the distribution of values for each measure.	Positive emotional well-being	Cantril ladder	Confounders: individual level: Individual socio-economic status (SES) and perceived neighbourhood safety area level: neighbourhood aesthetics, neighbourhood SES, and urban/rural geographic location. moderator: Age, gender, ethnicity, urban/rural geographic location	There was a non-significant linear trend observed for the overall relationship with green space and positive emotional well-being.
8	Li et al. 2018 (United States) [28]	Cross-sectional study	*n* = 155, high school students	Pearson’s pairwise correlations Multilevel modelling (MLM)	Concentration of nature on the point locations	Google Street View provides panoramic and omnidirectional views of street scenes, calculate the density of vegetation objectively in each scene	Mood	Adapted the Profile of Mood States questionnaire, 2nd Edition–Youth (POMS-Y)	Individual confounders: gender, age, SES (parental income, parental education attainment and parental occupation), race/ethnicity, automobile access and dog ownership day-by-day (DBD) level confounders: day of week, total free time, total TV time, total physical activity time, and number of activities during the day	The concentration of nature was associated significantly and negatively with depression (r = −0.09, *p* < 0.05), anger (r = −0.16, *p* < 0.01), fatigue (r = −0.12, *p* < 0.01), overall mood (*n* = −0.13, *p* < 0.01), and mood disturbance (B = −0.22, *p* < 0.05). Adolescents who spent more time outdoors were marginally more likely to have fewer mood disturbances (B = −0.02, *p* < 0.1). this relationship did not vary by demographic or SES.
9	Li et al. 2016 (United States) [32]	Randomized Controlled Trial (RCT)	*n* = 94, high school students	ANOVA	Windows opened on to green space	NA	Stress	Subjective stress: Visual Analogue Scale (VAS) Objective stress: Electrocardiography (EKG), Blood Volume Pulse (BVP), Skin Conductance Level (SCL) and body temperature (BT)	Confounders: age, gender, race, grade, health information, self-reported chronic stress levels, self-reported chronic mental fatigue, and preference for their school landscape	Students’ stress levels increased during the class activities and decreased after the break. there was no significant difference in stress across the window view conditions ((F1, 84(treatment) = 1.93, *p* = 0.15, β2 = 0.04). Demographic factors, chronic stress, chronic mental fatigue, and preference explained 10% of the variation in stress reduction at the end of the break. After adding in classroom window condition, the model significantly improved (*p* < 0.05) and explained 17 percent of the total variance. Stress reduction in a green condition was 1.36 units higher than that of the barren condition. But the comparison between no window and barren conditions was only marginally significantly different (*p* = 0.07)
10	Mueller et al. 2019 (England and Wales) [55]	Cross-sectional study in a longitudinal study	*n* = 3683 aged 10–15	Linear regression models	Neighbourhood greenspace	The percentage of greenspace within each uk ward	Mental health and behaviour	Strengths and Difficulties Questionnaire (SDQ)	Covariates: gender, age in years, education of the mother (university degree or not), and ethnicity.	Fear of crime is a predictor of emotional symptoms, conduct problems, hyperactivity and inattention, peer relationship problems, and total difficulties, not green space. Neighbourhood deprivation was positively associated with conduct problems and peer relationship problems. ‘like living in neighbourhood’ was predicted to conduct problem, hyperactivity, inattention, and total difficulties.
11	Wang et al. 2019 (United States) [56]	Cross-sectional study	*n* = 4538, aged 12–17 years	Logistic regression models	Level of greenness surrounding residential area	NDVI values within 8 different buffer sizes, ranging from 250 m to 950 m at an increment of 100 m.	Serious psychological distress	Kessler 6 (K6) scale	Covariates/confounders: age, race/ethnicity, sex, health status, household income, educational level, urban/rural status, length of residence, year of CHIS*, and neighbourhood poverty level Confounders in sensitivity analyses: air pollution burden, obesity, smoking and Alcohol use Mediation: social cohesion	An inter-quartile increment of NDVI in 350 m buffer predicted decreased odds of SPDs by 36% in teens (OR = 0.64, 95% CI = [0.46, 0.91]). the NDVI-SPD associations remained almost unchanged (OR = 0.66, 95% CI = [0.47, 0.94]) for teens after further inclusion of social cohesion
12	Wallner et al. 2018 (Vienna) [48]	Cross-over field experiment	*n* = 60, aged 16–18 years	Kolmogorov-Smirnov tests with Lilliefors corrected p-values Mauchly’s test and Box M tests	Three different settings (inner urban small and heavily used park with a few trees and surrounded by heavily used streets and dense residential areas, a larger park with some tree clumps, or a larger broadleaved forest with some scattered meadows and low visitor numbers)	NA	Momentary mood state	The self-condition scale by Nitsch (readiness for action, readiness for exertion, alertness, state of mood, tension/relaxa-tion, and recuperation)	NA	State of mood was almost highest after the stay in the green spaces, declined in the classroom on average by 0.57 stanine units after stays in the small urban park, and by 0.67 units after stays in the large urban park, while this decline was much less expressed after stays in the forest (0.14 stanine units, *p* < 0.001).
13	Ward et al. 2016 (New Zealand) [29]	Cross-sectional study	*n* = 118, intermediate schools students aged 11–14 years	Generalised linear mixed model (GLMM)	Publicly accessible parks, sports fields, and reserves	GIS mapping of the GPS data against the parks dataset available through Open Street Map identify the time points, the percentage of total data points inside green spaces for each participant was calculated.	Emotional well-being	The Life Satisfaction Scale (LSS) derived from Hubener’s Student Life Satisfaction Scale Ten Domain Index of Well-being (TDIW) Happiness with life as a whole (HS)	Covariates: sex, age, school Mediator: Moderate-to-vigorous physical activity”	There were positive relationships between the proportion of time spent in green space and all three measures of emotional well-being (LSS β = 0.861, *p* < 0.001; TDIW β = 3.176, *p* < 0.001; HS β = 0.445 *p* < 0.001). Fixed effect of MVPA and green space on emotional well-being were reduced but still significant (LSS β = 0.661, *p* < 0.001; TDIW β = 2.670, *p* < 0.001; HS β = 0.363, *p* < 0.001)
14	Herrera et al. 2018 (Germany) [49]	Cross-sectional study in a cohort study	*n* = 2690, aged 16–18 years	Generalised estimating equations (GEE) models	Greenness of the home environment	An average NDVI was obtained using a 30 m by 30 m resolution in a 500 m radius around home addresses	Job-related stress	Trier Inventory for Chronic Stress (TICS)	Covariates: Sociodemographic (sex, highest educational status), non-job-related chronic stress, current status and job type, environmental covariates (distance to sports facilities, distance to nearest urban green space) Mediation: physical activity”	Prevalence of high levels of work discontent and work overload decreased by increasing level of greenness in a buffer of 500 m around the home

## Supplementary Materials

The following are available online at https://www.mdpi.com/1660-4601/17/18/6640/s1, Table S1: PRISMA check list, Table S2: Database search information, Table S3: Quality assessment tool, Table S4: Methodological quality, Table S5: Strength of the evidence.
