# Supplementary material for: The Association between Green Space and Adolescents’ Mental Well-Being: A Systematic Review
Source: Int J Environ Res Public Health. 2020 Sep 11;17(18):6640. doi: 10.3390/ijerph17186640 (PMC7557737; doi:10.3390/ijerph17186640)
Supplement: Supplementary file 1 [file ijerph-17-06640-s001.pdf]

Supplementary documents

Table S1. PRISMA check list

Section/topic	#	Checklist item	Reported on page #
**TITLE**			
Title	1	Identify the report as a systematic review, meta-analysis, or both.	P1
**ABSTRACT**			
Structured summary	2	Provide a structured summary including, as applicable: background; objectives; data sources; study eligibility criteria, participants, and interventions; study appraisal and synthesis methods; results; limitations; conclusions and implications of key findings; systematic review registration number.	P1
**INTRODUCTION**			
Rationale	3	Describe the rationale for the review in the context of what is already known.	P2
Objectives	4	Provide an explicit statement of questions being addressed with reference to participants, interventions, comparisons, outcomes, and study design (PICOS).	P2
**METHODS**			
Protocol and registration	5	Indicate if a review protocol exists, if and where it can be accessed (e.g., Web address), and, if available, provide registration information including registration number.	P2
Eligibility criteria	6	Specify study characteristics (e.g., PICOS, length of follow-up) and report characteristics (e.g., years considered, language, publication status) used as criteria for eligibility, giving rationale.	P3
Information sources	7	Describe all information sources (e.g., databases with dates of coverage, contact with study authors to identify additional studies) in the search and date last searched.	P2
Search	8	Present full electronic search strategy for at least one database, including any limits used, such that it could be repeated.	P3
Study selection	9	State the process for selecting studies (i.e., screening, eligibility, included in systematic review, and, if applicable, included in the meta-analysis).	P3
Data collection process	10	Describe method of data extraction from reports (e.g., piloted forms, independently, in duplicate) and any processes for obtaining and confirming data from investigators.	P2-P3
Data items	11	List and define all variables for which data were sought (e.g., PICOS, funding sources) and any assumptions and simplifications made.	P2-P4
Risk of bias in individual studies	12	Describe methods used for assessing risk of bias of individual studies (including specification of whether this was done at the study or outcome level), and how this information is to be used in any data synthesis.	P4
Summary measures	13	State the principal summary measures (e.g., risk ratio, difference in means).	P4
Synthesis of results	14	Describe the methods of handling data and combining results of studies, if done, including measures of consistency (e.g., I^2) for each meta-analysis.	P3-P4

Section/topic	#	Checklist item	Reported on page #
Risk of bias across studies	15	Specify any assessment of risk of bias that may affect the cumulative evidence (e.g., publication bias, selective reporting within studies).	P3
Additional analyses	16	Describe methods of additional analyses (e.g., sensitivity or subgroup analyses, meta-regression), if done, indicating which were pre-specified.	NA
**RESULTS**			
Study selection	17	Give numbers of studies screened, assessed for eligibility, and included in the review, with reasons for exclusions at each stage, ideally with a flow diagram.	P4
Study characteristics	18	For each study, present characteristics for which data were extracted (e.g., study size, PICOS, follow-up period) and provide the citations.	P4
Risk of bias within studies	19	Present data on risk of bias of each study and, if available, any outcome level assessment (see item 12).	P4. P19
Results of individual studies	20	For all outcomes considered (benefits or harms), present, for each study: (a) simple summary data for each intervention group (b) effect estimates and confidence intervals, ideally with a forest plot.	P5-P18
Synthesis of results	21	Present results of each meta-analysis done, including confidence intervals and measures of consistency.	P5-P18
Risk of bias across studies	22	Present results of any assessment of risk of bias across studies (see Item 15).	P29
Additional analysis	23	Give results of additional analyses, if done (e.g., sensitivity or subgroup analyses, meta-regression [see Item 16]).	NA
**DISCUSSION**			
Summary of evidence	24	Summarize the main findings including the strength of evidence for each main outcome; consider their relevance to key groups (e.g., healthcare providers, users, and policy makers).	P29-32
Limitations	25	Discuss limitations at study and outcome level (e.g., risk of bias), and at review-level (e.g., incomplete retrieval of identified research, reporting bias).	P32
Conclusions	26	Provide a general interpretation of the results in the context of other evidence, and implications for future research.	P32
**FUNDING**			
Funding	27	Describe sources of funding for the systematic review and other support (e.g., supply of data); role of funders for the systematic review.	NA

From: Moher D, Liberati A, Tetzlaff J, Altman DG, The PRISMA Group (2009). Preferred Reporting Items for Systematic Reviews and Meta-Analyses: The PRISMA Statement. PLoS Med 6(7): e1000097. doi:10.1371/journal.pmed1000097

Table S2. Database search information

Database	Search terms		Articles identified
Medline (Ovid interface)	Green space	(green space* or greenspace* or greenness or greenery or green area* or greenway* or green belt* or green corridor* or natural environment* or open space* or park or parks or natur* space* or naturalness or garden* or playground or canopy or tree* or forest or forests or woodland* or green roof* or roof garden* or arboretum or urban nature).ti,ab,kw. OR (green adj2 space*).ti,ab,kw. OR Parks, Recreational/ OR Gardens/ OR Trees/ OR Forests/	663
	Mental well-being	(wellbeing or well being or wellness or emotional health or psychological health or mental health or mood or depress* or anxiety or stress or happiness or pleasure).ti,ab,kw. OR Mental Health/ OR Anxiety/ OR Stress, Psychological/ OR Happiness/ OR Pleasure/ OR Depression/	
	Adolescent	Adolescent/ OR (teen* or adolescen* or youth* or juvenile* or young people or young person or young adult* or high school* or secondary school* or senior school*).ti,ab,kw.	
Cochrane central register of controlled trials (Ovid interface)	Green space	Trees/ OR (green adj2 space*).ti,ab,kw. OR (green space* or greenspace* or greenness or greenery or green area* or greenway* or green belt* or green corridor* or natural environment* or open space* or park or parks or natur* space* or naturalness or garden* or playground or canopy or tree* or forest or forests or woodland* or green roof* or roof garden* or arboretum or urban nature).ti,ab,kw.	116
	Mental well-being	Stress, Psychological/ OR Mental Health/ OR Anxiety/ OR Depression/ OR Happiness/ OR Pleasure/ OR (wellbeing or well being or wellness or emotional health or psychological health or mental health or mood or depress* or anxiety or stress or happiness or pleasure).ti,ab,kw.	
	Adolescent	Adolescent/ OR (teen* or adolescen* or youth* or juvenile* or young people or young person or young adult* or high school* or secondary school* or senior school*).ti,ab,kw.	
EMBASE (Ovid interface)	Green space	(green adj2 space*).ti,ab,kw. OR recreational park/ OR canopy/ OR "tree"/ OR forest/ OR (green space* or greenspace* or greenness or greenery or green area* or greenway* or green belt* or green corridor* or natural environment* or open space* or park or parks or natur* space* or naturalness or garden* or playground or canopy or tree* or forest or forests or woodland* or green roof* or roof garden* or arboretum or urban nature).ti,ab,kw.	734
	Mental well-being	wellbeing/ OR mental health/ OR mood/ OR depression/ OR anxiety/ OR happiness/ OR stress/ OR pleasure/ OR (wellbeing or well being or wellness or emotional health or psychological health or mental health or mood or depress* or anxiety or stress or happiness or pleasure).ti,ab,kw.	
	Adolescent	adolescent/ OR juvenile/ OR (teen* or adolescen* or youth* or juvenile* or young people or young person or young adult* or high school* or secondary school* or senior school*).ti,ab,kw.	
PsycINFO (Ovid interface)	Green space	(green adj2 space*).ti,ab,id OR "Nature (Environment)"/ OR Playgrounds/ OR (green space* or greenspace* or greenness or greenery or green area* or greenway* or green belt* or green corridor* or natural environment* or open space* or park or parks or natur* space* or naturalness or garden* or playground or canopy or tree* or forest or forests or woodland* or green roof* or roof garden* or arboretum or urban nature).ti,ab,id	298
	Mental well-being	Well Being/ OR Mental Health/ OR Anxiety/ OR Stress/ OR Happiness/ OR Pleasure/ OR (wellbeing or well being or wellness or emotional health or psychological health or mental health or mood or depress* or anxiety or stress or happiness or pleasure).ti,ab,id	
	Adolescent	(teen* or adolescen* or youth* or juvenile* or young people or young person or young adult* or high school* or secondary	

		school* or senior school*).ti,ab,id	
CINAHL plus (EBSCO Interface)	Green space	(MH "Natural Environment") OR "green space*" or greenspace* or greenness or greenery or "green area*" or "greenway*" or "green belt*" or "green corridor*" or "natural environment*" or "open space*" or park or parks or "natur* space*" or naturalness or garden* or playground or canopy or tree* or forest or forests or woodland* or "green roof*" or "roof garden*" or arboretum or "urban nature" or "green N2 space*"	348
	Mental well-being	(MH "Mental Health") OR (MH "Wellness") OR (MH "Depression") OR (MH "Anxiety") OR (MH "Stress") OR (MH "Happiness") OR (MH "Pleasure") OR wellbeing OR "well being" OR wellness OR "emotional health" OR "psychological health" OR "mental health" OR mood OR depress* OR anxiety OR stress OR happiness OR pleasure	
	Adolescent	(MH "Adolescence") OR teen* or adolescen* or youth* or juvenile* or "young people" or "young adult*" or "young person" or "high school*" or "secondary school*" or "senior school*"	
SCOPUS	Green space	" (TITLE-ABS-KEY (""green space*"" OR greenspace* OR greenness OR greenery OR ""green area*"" OR ""greenway*"" OR ""green belt*"" OR ""green corridor*"" OR ""natural environment*"" OR ""open space*"" OR park OR parks OR ""natur* space*"" OR naturalness) AND PUBYEAR > 1999) OR (TITLE-ABS-KEY (garden* OR playground OR canopy OR tree* OR forest OR forests OR woodland* OR ""green roof*"" OR ""roof garden*"" OR arboretum OR ""urban nature"" OR ""green W/2 space*"") AND PUBYEAR > 1999) "	1728
	Mental well-being	TITLE-ABS-KEY (wellbeing OR "well being" OR wellness OR "emotional health" OR "psychological health" OR "mental health" OR mood OR depress* OR anxiety OR stress OR happiness OR pleasure) AND PUBYEAR > 1999	
	Adolescent	TITLE-ABS-KEY (teen* OR adolescen* OR youth* OR juvenile* OR "young people" OR "young person" OR "young adult*" OR "high school*" OR "secondary school*" OR "senior school*") AND PUBYEAR > 1999	
GEOBASE	Green space	found in GEOBASE for 2000-2020: (("green space*" or greenspace* or greenness or greenery or "green area*" or "greenway*" or "green belt*" or "green corridor*" or "natural environment*" or "open space*" or park or parks or "natur* space*" or naturalness or garden* or playground or canopy or tree* or forest or forests or woodland* or "green roof*" or "roof garden*" or arboretum or "urban nature" or "green NEAR/2 space") WN KY) AND (English WN LA)	2136
	Mental well-being	found in GEOBASE for 2000-2020: ((wellbeing OR "well being" OR wellness OR "emotional health" OR "psychological health" OR "mental health" OR mood OR depress* OR anxiety OR stress OR happiness OR pleasure) WN KY) AND (English WN LA)	
	adolescent	found in GEOBASE for 2000-2020: ((teen* or adolescen* or youth* or juvenile* or "young people" or "young person" or "young adult*" or "high school*" or "secondary school*" or "senior school*") WN KY) AND (English WN LA)	

Table S3. Quality assessment tool

Item	Description	Scale
Methodological quality		
1. Study Design	Was the study design clearly identified, and is the rationale for choice of design evident?	1: The design was appropriate for the study question
		0.5: Insufficiently described
		0: The design was not appropriate for the study question
2. Population - Selection bias	Are the individuals selected to participate in the study likely to be representative of the target population?	1: Likely to be representative
		0.5: Insufficiently described
		0: Unlikely to be representative
3. Population –Inclusion bias	Is there evidence of bias in the percentage of selected individuals who provided data for inclusion in the analysis?	1: No evidence of bias
		0.5: Insufficiently described
		0: Evidence of bias
4. Green space measure - derivation	Was derivation of the green space variable well described?	1: Derivation of green space measure well described
		0.5: Insufficiently described
		0: Derivation of green space measure not described
5. Green space measure - type	Did the green space measure include information on type of green space?	1: Green space measure included information on type of green space
		0.5: Insufficiently described
		0: Green space measure did not include information on type of green space
6. Use of green space	Use of green space was measured and included in analysis	1: Measured use of green space
		0.5: Insufficiently described
		0: Did not measure use of green space
7. Statistical methodology	Was an appropriate statistical methodology used?	1: Evidence of appropriate methodology
		0.5: Insufficiently described
		0: No evidence of appropriate methodology

8. Outcome - reliable	Were the outcome measures reliable	1: gave the same information over different situations
		0.5: Insufficiently described
		0: gave the different information over different situations
9. Outcome - valid	Were the outcome measures valid?	1: the measure assessed what it was intended to measure.
		0.5: Insufficiently described
		0: the measure did not access what it was intended to measure.
10. Effect size	Was an effect size (estimate and confidence interval/standard error) reported for green space variable?	1: Effect size reported for green space
		0.5: Insufficiently described
		0: Effect size not reported for green space
11.Level of analysis	Was analysis of green space access in relation to outcome carried out at individual level or at ecological (area) level	1: Individual level
		0.5: Insufficiently described
		0: Ecological level

Table S4. Methodological quality

items	Weeland	Bezold	Feng	Feda	Greenwood	Gubbels	Huynh	Li 2018	Li 2016	Mueller	Wang	Wallner	Ward	Herrera	SUM
1. Study Design	1	1	1	1	1	1	1	1	1	1	1	0	1	1	13
2. Population - Selection bias	1	1	1	1	0.5	1	1	1	0.5	1	1	0.5	1	1	12.5
3. Population –Inclusion bias	0	1	0.5	0.5	1	0.5	1	0.5	1	0.5	0.5	1	0.5	0.5	9
4. Green space measure - derivation	1	1	1	1	1	1	1	1	1	1	1	1	1	1	14
5. Green space measure - type	0.5	0	0.5	1	0	0	1	0	0	0.5	0	1	1	0	5.5
6. Use of green space	0	0	0	0	0	1	0	0	0	0	0	0	0	0	1
7. Statistical methodology	1	1	1	1	1	1	1	1	1	1	1	1	1	1	14
8. Outcome - reliable	1	1	1	1	1	1	1	1	1	1	1	0.5	1	0.5	13
9. Outcome - valid	1	1	1	1	1	1	1	1	1	1	1	0.5	1	1	13.5
10. Effect size	1	1	1	1	1	1	1	0.5	1	1	1	1	1	1	13.5
11.Level of analysis	0	0	1	0	1	1	1	1	1	0	0	1	1	0	8
SUM	7.5	8	9	8.5	8.5	9.5	10	8	8.5	8	7.5	7.5	9.5	7	

Table S5. Strength of the evidence

Strength of association between greenspace and mental well-being indicator	Weeland	Bezold	Feng	Feda	Greenwood	Gubbels	Huynh	Li 2018	Li 2016	Mueller	Wang	Wallner	Ward	Herrera
1: Positive relationship, judged as a statistically significant positive relationship (using significance threshold $p<0.05$) after adjustment for confounders, with 'positive' defined as mental well-being promoting		√		√				√	√		√	√	√	√
2: Inconsistent relationship, judged as inconsistent results across different green space and mental well-being measures, with some statistically significant positive relaitonships			√		√									
3: Equivocal relationship, judged as a marginally statistically significant result or														

inconsistent results presented in the paper (for example, different results across sub-groups).														
4: No evidence of a relationship, judged as no statistically significant relationship in results.	√					√	√			√				
5: Negative relationship, judged as a statistically significant negative relationship (using significance threshold p<0.05) after adjustment for confounders, where ‘negative’ is defined as health demoting														
